# Retinoids in the visual cycle: role of the retinal G protein-coupled receptor

**DOI:** 10.1194/jlr.TR120000850

**Published:** 2021-02-06

**Authors:** Elliot H. Choi, Anahita Daruwalla, Susie Suh, Henri Leinonen, Krzysztof Palczewski

**Affiliations:** 1 Department of Ophthalmology, Gavin Herbert Eye Institute, Center for Translational Vision Research, University of California, Irvine, CA, USA; 2 Department of Pharmacology, Case Western Reserve University, Cleveland, OH, USA; 3 Departments of Physiology and Biophysics, and Chemistry, University of California, Irvine, CA, USA

**Keywords:** vision, vitamin A, retina, retinal pigment epithelium, visual pigments, visual chromophore, photoisomerase, retinal pigment epithelium-retinal G protein-coupled receptor opsin, CDHR1, cadherin-related family member 1, CRALBP, cellular retinaldehyde-binding protein, ERG, electroretinography, *Gnat1*^−/−^, *Gnat1* knockout, GPCR, G protein-coupled receptor, IRBP, interphotoreceptor retinoid-binding protein, LRAT, lecithin:retinol acyltransferase, RALBP, retinal-binding protein, RDH5, 11-*cis*-retinol dehydrogenase 5, RDH10, retinol dehydrogenase 10, RE, retinyl ester, RGR, retinal pigment epithelium-retinal G protein-coupled receptor, *Rgr*^−/−^, *Rgr* knockout, RPE, retinal pigment epithelium, RPE65, retinal pigment epithelium-specific 65 kDa

## Abstract

Driven by the energy of a photon, the visual pigments in rod and cone photoreceptor cells isomerize 11-*cis*-retinal to the all-*trans* configuration. This photochemical reaction initiates the signal transduction pathway that eventually leads to the transmission of a visual signal to the brain and leaves the opsins insensitive to further light stimulation. For the eye to restore light sensitivity, opsins require recharging with 11-*cis*-retinal. This *trans*-*cis* back conversion is achieved through a series of enzymatic reactions composing the retinoid (visual) cycle. Although it is evident that the classical retinoid cycle is critical for vision, the existence of an adjunct pathway for 11-*cis*-retinal regeneration has been debated for many years. Retinal pigment epithelium (RPE)-retinal G protein-coupled receptor (RGR) has been identified previously as a mammalian retinaldehyde photoisomerase homologous to retinochrome found in invertebrates. Using pharmacological, genetic, and biochemical approaches, researchers have now established the physiological relevance of the RGR in 11-*cis*-retinal regeneration. The photoisomerase activity of RGR in the RPE and Müller glia explains how the eye can remain responsive in daylight. In this review, we will focus on retinoid metabolism in the eye and visual chromophore regeneration mediated by RGR.

Vitamin A is an essential fat-soluble micronutrient that has different functional roles in the human body. By definition, vitamin A is all-*trans-*retinol; however, the term vitamin A is often used to refer collectively to the derivatives of all-*trans-*retinol, including retinyl esters (REs), retinaldehyde, and retinoic acid. It is well documented that vitamin A is essential for human survival at every stage of life. It has critical functions during embryogenesis, the development of organs, reproduction, immunity, and vision (1, 2, 3, 4, 5, 6, 7, 8). Besides these important roles, new physiological functions of vitamin A are continuously being discovered in the fields of neuroscience and lipid research (9, 10). Perhaps in recognition of these facts, the role of vitamin A in various organ systems has been an exciting area of research for decades. Most notably, the eye requires vitamin A and its derivatives for the maintenance of its function throughout the entire life cycle. Thus, a considerable number of studies has been devoted over past decades to our understanding of the complex biochemical processes that make vision possible. This process requires the absorption of vitamin A from the systemic circulation into the retinal pigment epithelium (RPE) lying adjacent to the photoreceptor layer of the retina. Thus, vitamin A deficiency or mutations in the enzymes involved in the retinoid cycle result in a broad spectrum of retinal diseases.

## Absorption of dietary vitamin A

One of the characteristics of vitamin A is that it cannot be synthesized by the human body and therefore must be obtained from a dietary source. Dietary vitamin A is mainly available in two forms, preformed vitamin A and provitamin A. Preformed vitamin A is generally found in animal sources including liver, eggs, fish, and dairy products. Conversely, provitamin A carotenoids are abundant in fruits and vegetables. The majority of preformed vitamin A consists of long-chain fatty acid esters of retinol, particularly REs. These esters are hydrolyzed into retinols by pancreatic lipase in the duodenum or phospholipase B at the brush border of enterocytes, and the resulting retinols are taken up by the enterocytes (11, 12, 13). Although each individual has a different extent of conversion efficiency, approximately half of provitamin A carotenoids are converted into retinol in the human intestine and then absorbed.

The retinol derived either from preformed vitamin A or provitamin A carotenoids can be reesterified to REs by an enzyme called lecithin:retinol acyltransferase (LRAT) (14, 15, 16). Then, the majority of REs are incorporated into the lipid core of the chylomicrons and further distributed to other tissues through the circulation. The chylomicrons are metabolized into chylomicron remnants by lipoprotein lipase. The chylomicron remnants containing most of the REs are then directed mainly to the liver (17, 18). As a result of these processes, the liver serves as the main reservoir of vitamin A. The remaining REs in the circulation are taken up by peripheral tissues including the adipose, heart, muscle, and lungs (19).

## Transport of vitamin A into the eye

Depending on the demand, the stored REs in the liver are mobilized and secreted as retinol into the systematic circulation. Retinol mainly circulates in the blood as a complex with retinol-binding protein 4 and transthyretin (20, 21). In the eye, uptake of retinol from the choriocapillaris is mediated by the transmembrane cell-surface STRA6 receptor of the RPE, a pigmented monolayer of cells located between the photoreceptors and choroid (20, 22). STRA6 catalyzes the release of retinol from retinol-binding protein 4 and then transports retinol to the cytosol. Besides the retinol from the systemic circulation, retinol released from the photoreceptor outer segments is another source of retinoids to the RPE. Interphotoreceptor retinoid-binding protein (IRBP) mediates the transport of retinoids between the photoreceptors and RPE by first sequestering them in the interphotoreceptor matrix (23, 24). Then, retinol is incorporated into the metabolic pathway of RPE cells that regenerates visual chromophore through a series of enzymatic reactions (8). Because vitamin A is a critical source of substrate for the metabolic pathway in the eye, a deficiency of dietary vitamin A can be a leading cause of blindness.

## Retinoids in vision

The significance of vitamin A in visual physiology has been recognized since ancient times (25, 26). Although the Egyptians did not know about vitamin A, they treated a visual deficiency involving the retina and cornea with animal liver (26). A major breakthrough occurred in 1950 when the formation of the visual pigment rhodopsin from vitamin A metabolites and the opsin protein was experimentally demonstrated (27). Further progress in understanding the enzymatic reactions of visual chromophore regeneration was made possible by the successful application of advanced human genetics, animal models, structural biology, and pharmacology. Such studies demonstrated that the reactions of the retinoid cycle include Schiff base formation and hydrolysis, alcohol/aldehyde redox chemistry, esterification, and isomerization coupled to ester cleavage (8).

The first step of vision begins when 11-*cis*-retinal bound to rhodopsin or cone opsins is photoisomerized to all-*trans*-retinal (28, 29). The resulting all-*trans*-retinal is reduced to all-*trans*-retinol and transported to the RPE, where *trans*-to-*cis* isomerization occurs to regenerate the visual chromophore. The key enzyme responsible for this reaction is RPE-specific 65 kDa (RPE65) localized to the endoplasmic reticulum of RPE cells (30, 31, 32, 33, 34). The importance of RPE65 in visual function is underscored by the finding that more than 100 mutations in *RPE65* are associated with retinal diseases (35, 36). The inheritance patterns of these mutations include autosomal dominant and autosomal recessive forms. Without a functional RPE65, an adequate amount of visual chromophore cannot be generated to meet the demand for visual function.

Besides the retinoid cycle, an opsin homolog in the RPE and the Müller glia of the retina was identified as a potential isomerase contributing to visual chromophore regeneration in daylight conditions (37). This putative opsin homolog was first cloned from a bovine RPE cDNA library in 1993 and referred to as RPE-retinal G protein-coupled receptor (RGR) (38). The cloned sequence contained homologous features and sequence motifs consistent with the structure of a G protein-coupled receptor (GPCR) (38). Although biochemical studies of RGR demonstrated its role as a possible photoisomerase that counteracts the *cis*-*trans* photoisomerization by rhodopsin and cone opsins (37, 39), several early observations argued against its photoisomerase activity. For example, *Rpe65* knockout (*Rpe65*^−/−^) mice were unable to generate a detectable amount of visual chromophore under photic conditions despite the expression of RGR. Other findings indicated that RGR could stimulate RPE65 activity independent of light and increases all-*trans*-RE hydrolase activity (40, 41). Because the findings surrounding RGR were inconsistent, more thorough investigations were necessary to determine the photoisomerase activity of RGR and delineate its involvement in chromophore regeneration (42, 43).

In this review, we focus on the regeneration of visual chromophore by RGR under sustained light conditions. We begin with an introduction of retinochrome, an opsin that is phylogenetically related to RGR. The dual photoisomerase system regulated by rhodopsin and retinochrome in invertebrates provides insight into the role of RGR in the human eye (44, 45). Following this, we summarize the enzymatic activities contributing to the retinoid cycle and the RGR-mediated pathway. Lastly, we discuss the function of RGR in vivo, localization of RGR, and other enzymes involved in the retinoid cycle.

## The evolutionary history of retinochrome and RGR

Opsins include a functionally diverse group of seven transmembrane domain receptors that covalently bind retinal as a chromophore. A comprehensive understanding of the opsins is paramount to our understanding of vision because opsins are essential molecules mediating phototransduction. Therefore, it is not surprising that studies on opsins and the visual system have evolved in parallel. In 1983, the bovine rhodopsin gene was first cloned and sequenced as an opsin gene (46). Subsequently, the bovine rhodopsin crystal structure was solved in 2000, providing the first GPCR structure to the scientific community (29). Due to these original works, bovine rhodopsin has served as a model system for investigating the function of opsins and GPCRs. Moreover, the evolutionary history of the opsins has been a topic of great interest since the first opsin gene sequence and structure became available. Today, there are more than 1,000 sequences of opsins available, from jellyfish to humans (47, 48). Sequence analysis of different opsins has enabled the identification of conserved sites. These highly conserved sites are presumed to be critical for function, whereas sequence differences between subgroups often reflect adaptive changes.

**Fig. 1:**
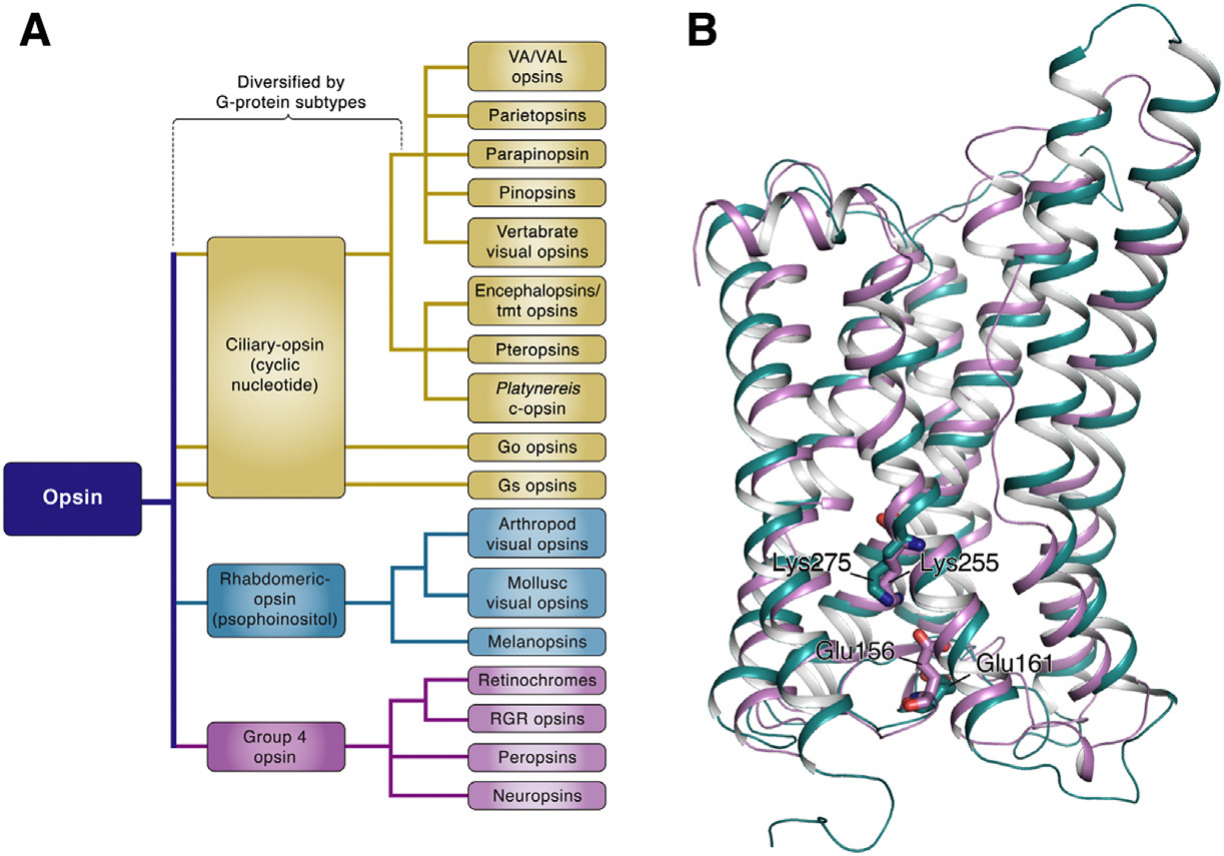
The phylogenic relationship of opsins and the structural relationship of retinochrome and RGR. A: Opsins are subdivided into three large groups including ciliary opsins, rhabdomeric opsins, and group 4 opsins. In ciliary photoreceptors, phototransduction is mediated by ciliary visual opsin, which uses a G protein specifically G_t_-mediated signaling cascade and elicits a hyperpolarization to light. In rhabdomeric photoreceptors, phototransduction is mediated by rhabdomeric opsin, which uses a G_q_-mediated signaling cascade and elicits a depolarization to light. Retinochrome and RGR, which are the group 4 opsins, act as photoisomerases. However, a G-protein cascade associated with the activation of retinochrome or RGR remains possible. B: Homology models of RGR from *Bos taurus* (violet) and retinochrome from *Todarodes pacificus* (teal blue) were created using Swiss-model (111). The proposed seven-transmembrane domain models of RGR and retinochrome were superimposed in PyMol editor. The chromophore-binding site at a Lys residue and the counterion Glu residue are highlighted as sticks.

Opsins can be categorized into three large groups: ciliary opsins, rhabdomeric opsins, and group 4 opsins (Fig. 1A) (47, 49). The ciliary opsins are mostly present in vertebrates and are characterized by their expression in ciliary photoreceptor cells and involvement in cyclic nucleotide signaling cascades. On the other hand, the rhabdomeric opsins are more commonly found in the rhabdomeric photoreceptor cells of invertebrates, except melanopsins, which are also expressed in vertebrates. In contrast to ciliary opsins, rhabdomeric opsins transmit light signals through phosphoinositol signaling mediated by a G protein called Gq. The last group, group 4 opsins, is comprised of relatively poorly characterized opsins including neuropsin, peropsin, retinochrome, and RGR. More recently, these group 4 opsins, particularly retinochrome and RGR, have gained increasing attention as their significance in visual physiology has surfaced (42, 43).

The retinochrome was first discovered in the squid retina in 1965 (50). The finding of retinochrome was particularly intriguing as it has different molecular properties from those of other opsin proteins. Unlike rhodopsin and cone opsins that photoisomerize 11-*cis*-retinal to all-*trans*-retinal, retinochrome preferentially binds all-*tran*s-retinal in the dark and isomerizes it to 11-*cis*-retinal in the light (44, 51, 52). Therefore, the retinochrome was postulated to generate 11-*cis*-retinal for regeneration of rhodopsin in cephalopods. In 1993, a homolog of retinochrome, RGR, was cloned from the mammalian retina (38). Based on phylogeny and intron positions, retinochrome and RGR clearly comprise a subclade, distinct from other opsins in the family (48).

The overall amino acid sequence of bovine RGR is 23.5% identical and 38.2% similar to that of the squid retinochrome. A region of RGR that encompasses the transmembrane domains IV and V and the connecting loop, from amino acid residues 139 to 189, is 45.1% identical and 66.7% similar to the corresponding region of retinochrome. Moreover, the seventh transmembrane domains of retinochrome and RGR contain a centrally positioned hydrophilic Lys residue (Fig. 1B). The retinal chromophore binds to this Lys residue through a protonated Schiff-base linkage (42, 53). The comparison of amino acid sequences also demonstrated that retinochrome and RGR lack a Glu residue at position 113, which serves as a counterion in vertebrate rhodopsin. However, Glu residues at positions 156 and 161, located in the extracellular IV-V loop, are believed to play this role in RGR and retinochrome, respectively (Fig. 1B). A site-directed mutagenesis study demonstrated that Glu 161 acts as the counterion in retinochrome (54). The study also suggested that the Glu residue at position 156 in RGR could serve as the counterion. Retinochrome and RGR also share other similarities that are lacking in other visual pigments. Retinochrome and RGR do not have putative glycosylation sites in the N terminus, and both have a relatively short C terminus (38). Taken together, this evidence suggests that RGR and retinochrome are evolutionarily and functionally similar to each other.

## The rhodopsin-retinochrome system in cephalopod photoreceptors

**Fig. 2:**
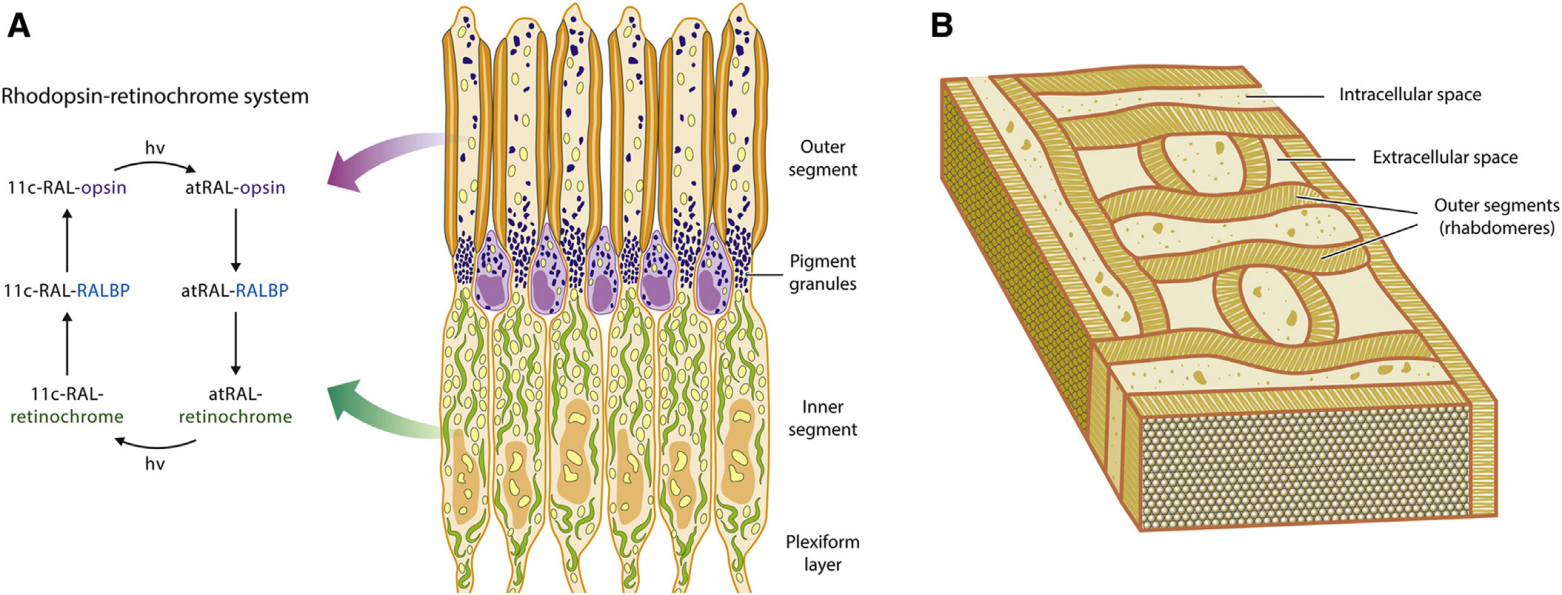
Rhodopsin-retinochrome system within the cephalopod photoreceptors and the three-dimensional reconstruction of the rhabdomeric membranes. A: Schematic representation of a longitudinal section through the cephalopod retina shows the outer segment, pigment granules, inner segment, and plexiform layer. The visual pigment, rhodopsin, is localized in the rhabdomal membranes of the outer segments. The other pigment, retinochrome, is localized in the myeloid bodies of the inner segments. The rhodopsin-retinochrome system maintains the visual sensitivity of the cephalopod retina. The soluble protein, RALBP, shuttles retinoids between rhodopsin and the retinochrome. B: Three-dimensional reconstruction of the outer segments of the cephalopod photoreceptors (112, 113). The outer segments possess two sets of stacked microvilli on opposite sides called rhabdomeres. The rhabdomeres of one cell sit orthogonally to the rhabdomeres of another cell, forming a lattice-like pattern.

At first glance, the exterior structures of the cephalopod eye look very similar to those of vertebrates. However, the cephalopod retina is everted in a way that the photoreceptor outer segments are arrayed anteriorly (55, 56). Therefore, the photoreceptor outer segments lie directly behind the lens and point toward incoming light. Posterior to the outer segments, black pigment granules form a layer that divides the outer segments and inner segments of the photoreceptors (Fig. 2A). Several lines of evidence have demonstrated that the pigmented layer migrates toward the outer segments in order to prevent excessive bleaching (57). The pigmented layer also serves as a line of demarcation between rhodopsin and retinochrome. Rhodopsin is located in the rhabdomal membranes of the outer segments, and retinochrome is localized in the inner segments (Fig. 2A, B) (44). Particularly, the myeloid bodies of the inner segments comprised of smooth membranes are enriched in retinochrome.

As in vertebrate photoreceptors, light causes isomerization of the visual chromophore to all-*trans*-retinal, thereby generating the visual signal (Fig. 2A). The all-*trans*-retinal dissociates from rhodopsin and then binds to retinal-binding protein (RALBP), which serves to shuttle the all-*trans*-retinal to retinochrome (58, 59). Then, retinochrome photoisomerizes the all-*trans*-retinal to 11-*cis*-retinal, which in turn is again carried by RALBP to metarhodopsin (58). Because rhodopsin in the rhabdomal membranes is located too far from retinochrome in the myeloid bodies to contact each other, RALBP plays an essential role in the rhodopsin-retinochrome system by shuttling the different retinoids. Although the cephalopod retina does not utilize multiple enzymes expressed in two cell types for the regeneration of the visual chromophore, as occurs in the vertebrate eye, the rhodopsin-retinochrome system nonetheless provides important evolutionary insight into the function of RGR, which is a close homolog of retinochrome.

## The retinoid (visual) cycle in vertebrate vision

**Fig. 3:**
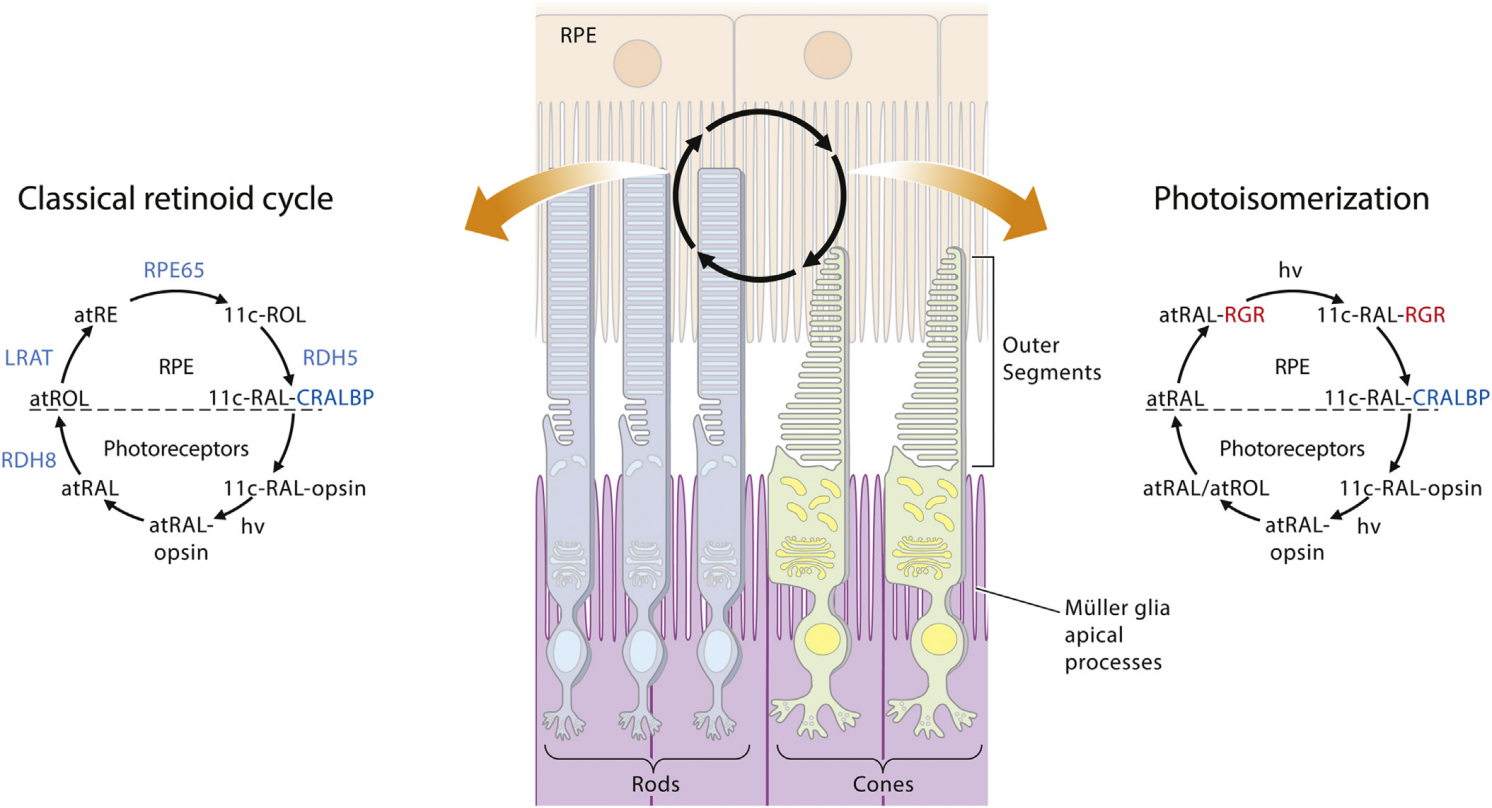
Photic chromophore regeneration mediated by RGR in the RPE. The classical retinoid (visual) cycle regenerates 11-*cis*-retinal through a series of enzymatic steps independent of light. At a critical point of the retinoid cycle lies RPE65, which isomerizes all-*trans*-REs to 11-*cis*-retinol. Through a photoisomerization cycle RGR further contributes to the regeneration of 11-*cis*-retinal upon illumination. CRALBP binds to 11-*cis*-retinal generated from either pathway and protects it from re-isomerization. The resulting 11-*cis*-retinal is transported to the rods and cones to regenerate rhodopsin and cone opsins, respectively. The isomerase (RPE65) and the photoisomerase (RGR) act in concert to maintain vertebrate vision.

Visual perception in humans is initiated in the photoreceptors of the retina, specifically in the outer segments of photoreceptors, when light enters the eye and activates the visual pigments (60, 61). Upon the absorption of a photon, 11-*cis-*retinal undergoes photoisomerization to all-*trans*-retinal, which induces a conformational change in the opsin protein and triggers the phototransduction cascade that ultimately leads to visual sensation (61, 62). To sustain normal visual sensitivity, all-*trans*-retinal must be converted back to 11-*cis*-retinal via the series of enzymatic reactions comprising the retinoid cycle, which occurs in a two-cell system comprised of photoreceptors and the RPE (Fig. 3) (8).

Here, we will first discuss the retinoid cycle in detail. When light isomerizes 11-*cis-*retinal to all-*trans-*retinal, the all-*trans*-retinal dissociates from the activated opsin (63, 64) and traverses one of two pathways. As soon as all-*trans-*retinal is released into the cytoplasm, it is reduced to all-*trans-*retinol by all-*trans*-retinol dehydrogenase 8 expressed in the outer segments of photoreceptors (65, 66, 67). However, all-*trans-*retinal can alternatively react with PE present in the disc membranes, forming a complex called N-retinylidine-PE within the lumen of the disc membrane (68). The resulting N-retinylidine-PE is then transported to the cytoplasmic disc surface by a membrane protein known as ABCA4 (68, 69) and reduced into all-*trans*-retinol by all-*trans*-retinol dehydrogenase 8 (70). All-*trans*-retinol exits the photoreceptor and enters the RPE, where the remaining retinoid cycle takes place. The transport of all-*trans-*retinol from the outer segments of photoreceptors to the RPE is facilitated by binding to IRBP (23, 24).

Once all-*trans-*retinol enters the RPE, it is esterified by LRAT to generate REs (16, 71, 72). These esters are the primary storage form of retinoids in retinosomes (73, 74) and the substrate for the next step of the retinoid cycle (75). The next step involves the simultaneous hydrolysis and isomerization of all-*trans-*REs to yield 11-*cis*-retinol by RPE65 (33, 34, 76). As a final step, the 11-*cis-*retinol is then oxidized to 11-*cis-*retinal by *cis*-RDHs, the most important of which is 11-*cis*-retinol dehydrogenase 5 (RDH5) (77). Newly generated 11-*cis-*retinal is transported across the subretinal space by IRBP to the photoreceptor outer segments to regenerate photosensitive visual pigment.

Delineating the biochemical reactions comprising this classical retinoid cycle was made possible by research extending over a period of more than 150 years. However, the rate of visual chromophore regeneration via the retinoid cycle is insufficient to meet the demand for 11-*cis*-retinal required to maintain light sensitivity, especially during daylight conditions. It was therefore recognized that additional pathways complementary to the retinoid cycle must exist to counter the forward *cis*-*trans* isomerization and enable photoreceptor cells, especially cones, to maintain their sensitivity. More recently, these additional pathways to regenerate the visual chromophore in daylight conditions have been investigated by several laboratories.

## Biochemical properties of RGR and photic generation of 11-*cis*-retinal by RGR

The photoreceptors in the retina are known to express opsins, but a large body of evidence has shown that other tissues express visual opsins or nonvisual opsins as well. These tissues include the pineal gland, parapineal structure, hypothalamus, and skin (78, 79, 80, 81, 82). As previously mentioned, the RPE cells express nonvisual opsins including peropsin and RGR. The similarity of amino acid sequences between RGR and retinochrome initially suggested that RGR could function as a photoisomerase. In addition, the sequence analysis of bovine RGR identified the conserved Lys residue at position 255 that serves as the retinal-binding site in visual pigments. Indeed, RGR was shown to bind both all-*trans*-retinal and 11-*cis*-retinal, but the binding affinity was consistently higher with all-*trans*-retinal (83). Further studies demonstrated that immunopurified RGR from bovine RPE microsomes binds all-*trans*-retinal, and the addition of hydroxylamine could abolish the specific binding of all-*trans*-retinal to RGR by acting as a scavenger of retinal (42, 84).

An electron microscopy study with immunogold staining demonstrated that RGR was localized in the RPE and Müller glia of the bovine retina (85). RGR was localized predominantly to the smooth endoplasmic reticulum in the RPE, whereas it was localized to the end-feet and proximal processes of the Müller glia. This pattern of localization was consistent with the expression profile observed from the single-cell RNA sequencing of mouse, bovine, and human samples (42). However, a considerably low amount of *Rgr* transcripts was detected in mouse Müller glia compared with bovine and human Müller glia. In line with the low amount of Rgr transcripts in mouse Müller glia, the amount of RGR protein also was found to be very low in mouse Müller glia compared with that in mouse RPE (43).

Another study employed HPLC analysis to identify the endogenous retinoid that binds to RGR in the dark. The analysis revealed that all-*trans* isomer was the predominant retinal bound to RGR in the dark (39). Together, these findings provide compelling evidence that RGR specifically binds all-*trans*-retinal potentially through the Lys residue at position 255. Later, a site-directed mutagenesis study confirmed that the Lys residue at position 255 is essential for the Schiff base formation and function of RGR (42).

Additional understanding of the biochemical properties of RGR was achieved with its purification from bovine RPE microsomes or from a mammalian expression system (42, 84). The UV-visible light absorption spectrum of RGR bound to all-*trans*-retinal showed a peak around 470 nm, with little change observed upon illumination (42, 84). The 470 nm peak completely disappeared after incubation with hydroxylamine, which allows the hydrolysis of the Schiff base and formation of retinal oxime from the bound retinal (42). The omission of all-*trans*-retinal prior to solubilization and purification of RGR also substantially diminished the height of the 470 nm peak compared with that of RGR bound to all-*trans*-retinal (84). Increase in the pH value from 6.5 to 8.0 also significantly reduced the absorbance at 470 nm. The effect of the change in hydrogen ion concentration suggests that the protonation of the Schiff base is critical for the 470 nm peak. Also, this indicates that the p*K*_a_ of the Schiff base is close to 6.5. Consistent with the previous results demonstrating the binding of all-*trans*-retinal to RGR, the shape of the absorption peak and the spectral properties of immunopurified RGR also supported that RGR binds all-*trans*-retinal through a Schiff base formation. However, additional work was required to evaluate the function of RGR as a photoisomerase.

The first attempt to observe the photoisomerase activity of RGR was made in 1999 (39). Immunopurified bovine RGR was illuminated with 470 nm monochromatic light. Although the experiment demonstrated that RGR photoisomerizes all-*trans*-retinal to 11-*cis*-retinal, it was several years before RPE65 was identified as a retinoid isomerase (33, 34, 76), and on its own RGR activity was insufficient to prove that RGR could robustly generate 11-*cis*-retinal under photic conditions. Also, there was no direct evidence that the 11-*cis*-retinal from RGR dissociates and enters the pathway for regeneration of the visual pigments (39). Therefore, the function of RGR remained unclear for two decades.

The functional aspects of RGR were eventually revisited and provided more instructive information. For example, a precise illumination system with a narrow bandwidth allowed our group to determine the action spectrum for 11-*cis*-retinal production by RGR. Surprisingly, the highest synthesis of 11-*cis*-retinal by RGR occurred between 500 nm and 530 nm, which does not overlap with the absorption spectrum of RGR (42). This finding was consistent using cell lysates expressing RGR, immunopurified RGR, or bovine RPE microsomes (42). Differences between the absorption and action spectra likely arise from the multitude of competing photoisomerization reactions that occur in the complex mixture of proteins and membranes.

Within the RPE, cellular retinaldehyde-binding protein (CRALBP) serves as an acceptor of 11-*cis*-retinal. Purified CRALBP from the RPE binds only 11-*cis*-retinal, whereas purified CRALBP from the retina binds 11-*cis*-retinal and 11-*cis*-retinol in a ratio of 3:1 (86). Thus, it is reasonable to speculate that CRALBP could bind 11-*cis*-retinal formed by RGR and thereby protect the 11-*cis*-retinal from re-isomerization to all-*tran*s-retinal. Indeed, the addition of CRALBP to the RGR-mediated photoisomerization reaction increased 11-*cis*-retinal production 3.5-fold (42). Taken together, the proper wavelength of stimulating light and the presence of CRALBP were essential to demonstrate the robust photoisomerase activity of RGR. These findings explain in part why the photoisomerase activity of RGR seemed low when it was first evaluated in 1999. Also, these recent findings suggest that CRALBP could mediate 11-*cis*-retinal production by RGR in the RPE and visual pigment renewal in the photoreceptors (Fig. 3).

**Fig. 4:**
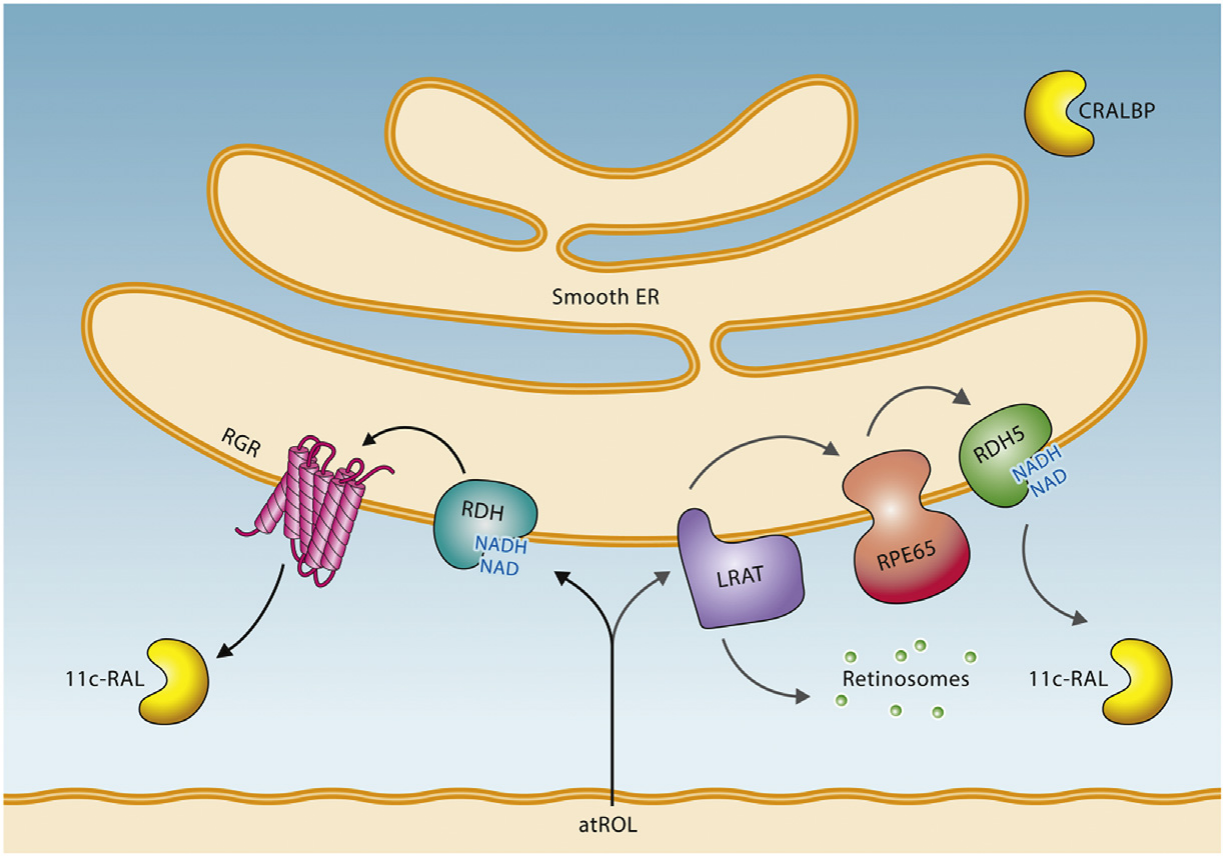
Flow of retinoids within the RPE. All-*trans*-retinol (atROL) from the photoreceptors is either oxidized to all-*trans*-retinal by RDH or esterified to all-*trans*-REs by LRAT. Thus, these reactions constitute a bifurcation during retinoid metabolism in the RPE. RGR binds the resulting all-*trans*-retinal and photoisomerizes it to 11-*cis*-retinal. RPE65 binds all-*trans*-REs and isomerizes them to 11-*cis-*retinol. As a final step, RDH5 oxidizes 11-*cis*-retinol to 11-*cis*-retinal. Retinosomes can store excess all-*trans*-REs. CRALBP transports the 11-*cis*-retinal generated from both pathways to the rod and cone outer segments to form active visual pigments.

Although these findings demonstrated that RGR could photoisomerize all-*trans*-retinal to 11-*cis*-retinal in a robust manner, it was unclear how RGR within the RPE gains access to all-*trans*-retinal, because the concentration of all-*trans*-retinal in the RPE is known to be very low (87). A previous study showed that bovine RPE cultured with radioactive all-*trans*-retinol could produce REs and all-*trans*-retinal in a ratio of 2.7 to 1 (88). Then, the resulting radioactive all-*trans*-retinal bound to RGR (88). This finding was further supported by the observation that bovine RPE microsomes could generate 11-*cis*-retinal upon light exposure in the presence of all-*trans*-retinol and NAD^+^ (42). Collectively, these findings demonstrated that all-*trans*-retinol is efficiently oxidized by RDH in the presence of the dinucleotide cofactor, and the product, all-*trans*-retinal, binds to RGR. These findings also indicate that the esterification of all-*trans*-retinol by LRAT and the oxidization of all-*trans*-retinol by RDH represent a bifurcation point for retinoid metabolism in the RPE (Fig. 4). Both reactions are critical for visual chromophore regeneration in that they provide the substrate for RPE65 and RGR.

The aforementioned studies primarily focused on RGR in bovine RPE microsomes and recombinant bovine RGR. Another recent study suggested that RGR in the Müller glia of the retina works in concert with retinol dehydrogenase 10 (RDH10) to achieve photic production of 11-*cis*-retinol (43). Human embryonic kidney 293T (HEK293T) cells transiently expressing both RGR and RDH10 produced 11-*cis*-retinol upon light exposure. In this experiment, the action spectrum of RGR activity overlapped with its absorption spectrum. The degree of 11-*cis*-retinol production with 470 or 500 nm light did not differ (43). Cumulatively, these recent studies demonstrate that RGR is responsible for photoisomerization of all-*trans*-retinal to 11-*cis*-retinal. They also demonstrate that the proteins acting in concert with RGR to carry out the light-dependent isomerization can differ between cell types (42, 43).

## Physiological role of RGR in the eye

RGR as a necessary element for continuous visual responsiveness in bright light was first described two decades ago (37). Chen et al. (37) kept *Rgr* knockout (*Rgr*^−/−^) and WT littermate mice at 4,000 lux luminance (bright daylight illuminance) for 8 h and subsequently analyzed the amount of 11-*cis*-retinal and rhodopsin in their eyes. The results showed that the amount of 11-*cis*-retinal and rhodopsin in *Rgr*^−/−^ mice was approximately half of their WT counterpart, whereas the amount of 11-*cis*-retinal and rhodopsin was comparable between the *Rgr*^−/−^ and WT mice after overnight dark adaptation. In addition, when researchers kept *Rgr*^−/−^ mice at 1,400 lux for 10 h, they accumulated over 10-fold more all-*trans*-REs than WT mice. These findings demonstrate that RPE65-mediated regeneration of visual chromophore occurred in the dark, whereas in conditions resembling daylight, RGR activity was required to augment the regeneration of visual chromophore to normal levels. Finally, no differences were observed between electroretinography (ERG) responses recorded in dark-adapted *Rgr*^−/−^ mice compared with WT mice. However, when the recordings were performed after 4 h light-adaptation (900 lux), the ERG amplitudes were significantly attenuated in the *Rgr*^−/−^ mice. These results are consistent with biochemical findings that RGR functions in preventing the saturation of photoreceptors under bright light conditions, and thus facilitates vision in daylight.

Humans and mice lacking retinoid isomerase RPE65 still have residual response to light (31, 89, 90, 91). The double knockout of RGR and RPE65 appears to eliminate the production of 11-*cis*-retinal (90). Further investigations regarding the function of RGR as a photoisomerase facilitating photopic vision were recently conducted by Morshedian et al. (43). They crossed *Rgr*^−/−^ mice with *Gnat1* knockout (*Gnat1*^−/−^) mice (43), which blocks phototransduction in rod cells and thereby allows the measurement of phototransduction activity solely in cones. They used a transretinal ex vivo ERG technique, whereby the neural retina was excised from the eye cup, perfused, and stimulated without interference from the remainder of the eye, including the RPE (43). Retinas excised from *Rgr*^−/−^/*Gnat1*^−/−^ or *Gnat1*^−/−^ mice were first subjected to dark-adapted ERG recording, and thereafter exposed to continuous ambient light for 60 min while the ERG responses were recorded at 15 min intervals (43). RGR-deficient retinas lost their sensitivity to light stimulation faster than retinas expressing RGR. Moreover, RGR-expressing retinas recovered 10-fold higher sensitivity compared with RGR-deficient retinas after a 30 s bright light exposure that was calculated to bleach 90% of the cone photopigment. The results clearly demonstrate that RGR is crucial for retaining cone photoreceptor sensitivity in daylight conditions. The authors proposed that the RGR-derived photoisomerization occurred in Müller glia. The argument was bolstered by experiments showing that WT mouse retinas treated with a Müller glia toxin diminished photopic ERG responses in a similar fashion as the RGR knockout. The interpretation of the data was simplified, because the ex vivo ERG is performed using retinas excised from the eye cup, therefore without a contribution from the RPE, including RPE65-mediated pigment regeneration. In summary, recent studies confirmed that RGR photoisomerizes 11-*cis*-retinal in vivo, and the evidence strongly suggests that RGR plays an important role in visual function in bright light conditions (42, 43).

Besides the physiological role of RGR identified in *Rgr*^−/−^ mice, a mutation of *RGR* implicated in human retinal dystrophies also suggests that RGR plays a significant role in human vision. In 1999, a homozygous Ser66Arg mutation and a heterozygous frameshift mutation in the RGR gene were associated with certain retinal dystrophies (92). Both mutations were reevaluated with modern genome sequencing technologies, such as whole-genome sequencing and next-generation sequencing (93, 94). Surprisingly, a frameshift mutation of cadherin-related family member 1 (*CDHR1*) was also found in the individuals carrying the Ser66Arg mutation (93). It is conceivable that the frameshift mutation of *CDHR1* is the cause of retinal degeneration in these individuals because mutations in *CDHR1* are associated with retinal dystrophies (100, 95, 96, 97, 98, 99). However, the frameshift mutation of *RGR* has been confirmed in individuals with retinal degeneration (94). Thus, findings to date support the conclusion that RGR is an essential enzyme in the human eye.

Pharmacological visual cycle modulators and genetic models added new insights about the mechanisms of cone photopic visual responses. Acute administration of potent RPE65 inhibitors did not affect the light sensitivity of cone photoreceptors in mice during extended exposure to background light. The cone function was only partially suppressed in cone-dominant ground squirrels, suggesting RPE65-independent regeneration mechanisms (101). In zebrafish, it was shown that RPE65 regenerates photopigment required by cones for immediate photopic response; however, during sustained light, a light-dependent regeneration of visual pigments appeared to be important to produce 11-*cis*-retinal levels sufficient for photopic vision (102).

## Potential interaction of RGR with enzymes involved in the retinoid cycle

For the most part, RGR has been examined as a singular enzyme engaged in a visual pathway consisting of other relatively separate enzymes. While this may be accurate, several lines of investigation point to the possibility of RGR acting as part of a complex and, therefore, its function and regulation cannot be fully appreciated in the absence of the other proteins and cofactors that form the complex.

Initial investigations in the 1990s determined that RGR was localized to the smooth endoplasmic reticulum of RPE cells and Müller glia of the bovine retina, and that this distribution coincided with the localization of two retinoid-binding proteins, CRBP and CRALBP (85). However, the role of RGR in the regeneration of the visual chromophore was not appreciated at the time, therefore the potential for RGR to physically interact with retinoid-binding proteins was not adequately investigated. Later, several studies of RGR and its interactions with enzymes in the visual cycle were more instructive.

In 2001, Fong and colleagues showed that RGR associated reproducibly with another protein when purified from bovine RPE (103). They identified the copurified protein as RDH5, the enzyme primarily known to be responsible for the oxidation of 11-*cis*-retinol to 11-*cis*-retinal in the presence of NAD^+^ or the reduction of 11-*cis*-retinal to 11-*cis*-retinol in the presence of NADH. Formation of 11-*cis*-retinol from 11-*cis*-retinal was noted when RPE microsomes containing RGR and RDH5 were illuminated in the presence of NADH, suggesting a reductase role for RDH5 in this reaction (42, 103). In addition, they observed an increase in the production of 11-*cis*-retinol accompanying this photoisomerization process. Although RGR was shown to photoisomerize all-*trans*-retinal to 11-*cis*-retinal previously, complete photoisomerization to and release of the *cis*-isomer was not seen (39). They suggested an additional reductase role for RDH5 in this coupled reaction that they deemed was essential for complete stereospecific photoisomerase activity of RGR in the RPE. However, the physiological relevance of this interaction has not been confirmed. Soon afterwards, an analysis of RPE microsomes indicated that CRALBP, RDH5, RPE65, and RGR tend to immuno-precipitate as a complex, with RPE65 interacting directly with RGR (104, 105).

Using *Rgr*^−/−^ mice, Wenzel et al. (40) then demonstrated an interdependence of RGR and RPE65. The expression of RPE65 protein in these mice was reduced by more than half in comparison to that seen in WT mice, which suggested a possible stabilizing interaction between the two enzymes. The subsequent identification of RPE65 as the visual cycle isomerase (33, 34) and the characterization of its crystal structure (106, 107, 108) revealed further details about the interactions of RGR and other RPE microsomal proteins with RPE65. To that end, it was shown that RGR copurified with RPE65 and RDH5 from RPE microsomes treated with a cross-linking reagent (108). Although they successfully identified a site of interaction between RPE65 and RDH5, they were unable to do the same for RGR and RPE65. Further investigations into the mode of interaction of RGR with RPE65 and RDH5 are needed to corroborate the existence of a multiprotein complex in the classical visual cycle in the RPE.

Unfortunately, little attention has been focused on the physical interactions of RGR with other visual cycle enzymes in Müller cells. Although the dependence of RGR activity on RDH10 has been established in glia cell culture (43), the presence of a direct association between these two enzymes has not yet been determined. Ultimately, understanding the interactions within a multiprotein complex consisting of RGR, RPE65, RDHs, and retinoid-binding proteins could help to explain why patients suffering from different types of retinal dystrophies, thought to be attributed to a deficiency in one of these proteins, can show either disparate or overlapping phenotypes (36, 94, 109, 110).

## Concluding remarks

Compared with the invertebrate eye, the vertebrate eye is more complicated because it involves multiple cell types, numerous enzymes, and the intercellular flow of retinoids facilitated by different binding proteins. However, this complex pathway allows the vertebrate eye to adapt to rapidly changing light conditions. In contrast, this cannot be achieved in the invertebrate eye because the bleaching rate of the visual pigments and the regeneration rate of visual chromophore in the photoreceptors are tied to the photon flux.

The critical role of the RPE in visual chromophore regeneration has been known for over two centuries. The retinoid cycle in the RPE provides a continuous supply of visual chromophore independent of light. In addition to the classical retinoid cycle, RGR in the RPE and Müller glia contributes to the production of visual chromophore, particularly in response to the level of light exposure. Because the isomerase, RPE65, and the photoisomerase, RGR, operate together, the vertebrate retina can sustain vision with an inexhaustible supply of visual chromophore in the dark or in different levels of illumination.

## Conflict of interest

The authors declare that they have no conflicts of interest with the contents of this article.
